# Kienböck's disease: a case report

**DOI:** 10.11604/pamj.2015.22.246.6837

**Published:** 2015-11-16

**Authors:** Youssef Omor, Ittimade Nassar, Ali Ajana, Nabil Moatassimbillah

**Affiliations:** 1 Department of Medical Imaging, Mohammed V University, URAC 30, Faculty of Medicine and Pharmacy, Rabat, Morocco

**Keywords:** Kienböck disease, lunate osteonecrosis, magnetic resonance imaging

## Abstract

Kienböck disease is a condition characterized by avascular necrosis of the lunate bone. Advanced imaging can aid in the diagnosis and staging of Kienböck disease. Magnetic resonance imaging (MRI) is an important adjunct to diagnosis. In particular, MRI is helpful early in the disease when plain radiographs may not reveal abnormalities. A 17 -year-old man with Kienböck disease who underwent radiography and MR is described in this article.

## Introduction

Kienböck disease is a condition marked by avascular necrosis of the lunate bone [1]. MRI is useful in diagnosis and staging and should be considered, after conventional radiography, for patients with suspected Kienböck disease [2]. There are few reports in children and therapeutic recommendations in the literature about this condition [3]. We report here a case of a 17-year-old male teenager.

## Patient and observation

**Figure 1:**
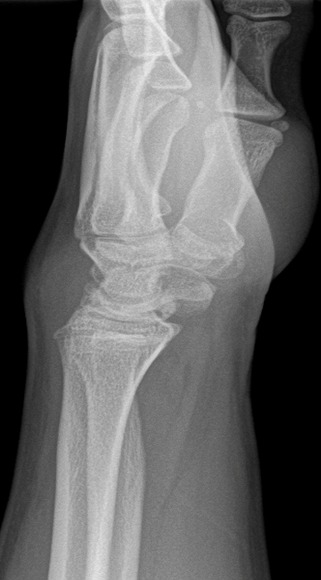
Lateral radiographs wrist showed a densification of the lunate with a flattened and irregular appearance of the lunate, without signs of osteoarthritis

**Figure 2:**
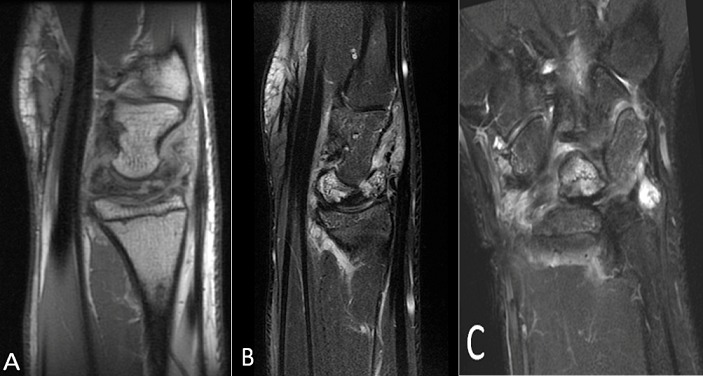
MRI of the left wrist: (A) sagital protons density showing collapse of the lunate with mixesd signal intensity; (B) sagital T2-weighted fat-suppressed MR image showing fragmented and irregular appearance of the lunate with hyper signal intensity; (C) coronal STIR showing fragmented and irregular appearance of the lunate with hyper signal intensity

A 17-year-old, left-handed, suffered progressive dorsal left wrist pain, six months before his consultation. No specific wrist injury was reported. The patient complained of a slight loss of the arc of wrist flexion/extension along with substantial loss of grip strength.. Clinical examination showed marked tenderness over the dorsal aspect of the wrist. Flexion/extension of the left wrist was 85°/85° compared with 90°/90° on the right side. Pronation and supination were normal. Grip strength was 10 kg on the dominant left side compared with 20 kg on the right.On standard PA and lateral radiographs wrist showed a densification of the lunate with a flattened and irregular appearance associated with a fixed rotation of the scaphoid, without signs of osteoarthritis.The diagnosis was made of stage III Kienböck's disease according to Lichtman's classification (Figure 1). An MRI was performed confirming the diagnosis by objectifying a fragmented and irregular appearance of the lunate with a revamped appearance on T2 and STIR associated with a thickening of the subcutaneous soft tissue and minimal effusion at the styloid recess (Figure 2). Surgical treatment option was refused by the patient and his parents. It was decided to immobilize the wrist in a splint and to stop sport activities the patient will be convened within 3 months for clinical and radiological control.

## Discussion

Kienböck's disease is rare in children, only a few cases have been published [3, 4]. It's most commonly affects men between the ages of 20 and 40 years [5]. The disease commonly affects the dominant wrist [2]. Many patients describe a history of trauma [5], but this is not always present as is the case of our patient.

Kienböck disease was described by the Austrian radiologist Robert Kienböck in 1910 as a condition characterized by avascular necrosis of the lunate bone. It is also known as osteonecrosis, lunatomalacia, and aseptic or ischemic necrosis of the lunate [2].

The pathophysiologic mechanism of this entity is multifactorial [2].There is no single definitive cause of Kienböck disease, a complex interplay of vascular and anatomic variations, combined with varying degrees of microtrauma and insults, contribute to its development [5].

The symptoms of Kienböck disease can vary depending on their stage at initial presentation, patients typically present with pain localized to the radiolunate facet, decreased motion, swelling, and weakness in the affected hand. Pain is classically insidious in onset, often related to activity, and can be present for extended periods before presentation [5].

Radiography is the initial imaging technique for assessing Kienböck disease and also can be used to rule out other pathologic conditions, such as arthrosis and fractures [2].Plain radiography allows the disease to be classified into 4 stages according to Lichtman and associates [6]. This classification is highly reliable and reproducible and has the most clinical relevance because it helps in determining the most appropriate treatment [2].

MRI is likely to be the next best imaging examination after routine radiography [2]. In the early stages of the disease, the use of MRI can aid in making the diagnosis and is more sensitive and specific than bone scanning [6]. Furthermore, MRI is useful for longitudinal assessment of the postoperative response to direct and indirect revascularization procedures. Contrast-enhanced MRI is important for determining the degree of necrotic tissue and the most appropriate treatment of stage II and IIIA disease. Contrastenhanced, MRI is not necessary in stages I, IIIB, IIIC, or IV because the degree of necrosis does not change treatment in these stages [2].

Kienböck disease remains a challenging clinical problem [1]. Kienböck disease is often a progressive disorder resulting in joint destruction within 3–5 years if untreated [2]. There remains no definitive treatment of this entity. There are several treatment options, largely based on the stage at presentation. Although options vary, they typically fall into several broad categories: unload the lunate, revascularize the lunate, or treat carpal instability and collapse with salvage procedures [5].

## Conclusion

Kienböck disease is a condition marked by avascular necrosis of the lunate bone. MRI can help in visualizing of the bone anatomy, the staging of Kienböck disease, and ruling out alternative diagnoses that mimic Kienböck disease (pseudo-Kienböck lesions). MRI therefore should be considered after conventional radiography in the care of patients with suspected Kienböck disease.

## References

[1] Kevin Lutsky, Pedro Beredjiklian K (2012). Kienböck Disease. J Hand Surg..

[2] Javier Arnaiz, Tatiana Piedra, Luis Cerezal, John Ward, Alex Thompson, Jorge Vidal A, Ana Canga (2014). Imaging of Kienböck Disease. AJR Am J Roentgenol.

[3] Guillaume Herzberg, Sylvie Mercier, Jean Pierre Charbonnier, Philippe Got (2006). Kienböck's Disease in a 14-Year-Old Gymnast: a Case Report. J Hand Surg.

[4] Luc De Smet (2003). Kienböck's disease in a 12-year-old girl. Acta Orthopædica Belgica.

[5] Danielle Cross, Kristofer Matullo S (2014). Kienböck ‘s Disease. Orthop Clin N Am.

[6] Richard Hurley T, Michael McKee D (2008). Kienböck's disease: an unusual cause of wrist pain in a 13-year-old girl. Can J Surg..

